# Learned spectral decoloring enables photoacoustic oximetry

**DOI:** 10.1038/s41598-021-83405-8

**Published:** 2021-03-22

**Authors:** Janek Gröhl, Thomas Kirchner, Tim J. Adler, Lina Hacker, Niklas Holzwarth, Adrián Hernández-Aguilera, Mildred A. Herrera, Edgar Santos, Sarah E. Bohndiek, Lena Maier-Hein

**Affiliations:** 1 Computer Assisted Medical Interventions, German Cancer Research Center, Heidelberg, Germany; 2 Medical Faculty, Heidelberg University, Heidelberg, Germany; 3 Institute of Applied Physics, Biomedical Photonics, Bern University, Bern, Switzerland; 4 Faculty of Mathematics and Computer Science, Heidelberg University, Heidelberg, Germany; 5 Faculty of Physics and Astronomy, Heidelberg University, Heidelberg, Germany; 6 Department of Neurosurgery, Heidelberg University Hospital, Heidelberg, Germany; 7 Department of Physics, University of Cambridge, JJ Thomson Avenue, Cambridge, CB3 0HE UK; 8 Cancer Research UK Cambridge Institute, University of Cambridge, Robinson Way, Cambridge, CB2 0RE UK

**Keywords:** Molecular imaging, Optical imaging, Computational science, Diagnostic markers

## Abstract

The ability of photoacoustic imaging to measure functional tissue properties, such as blood oxygenation sO$_2$, enables a wide variety of possible applications. sO$_2$ can be computed from the ratio of oxyhemoglobin HbO$_2$ and deoxyhemoglobin Hb, which can be distuinguished by multispectral photoacoustic imaging due to their distinct wavelength-dependent absorption. However, current methods for estimating sO$_2$ yield inaccurate results in realistic settings, due to the unknown and wavelength-dependent influence of the light fluence on the signal. In this work, we propose *learned spectral decoloring* to enable blood oxygenation measurements to be inferred from multispectral photoacoustic imaging. The method computes sO$_2$ pixel-wise, directly from initial pressure spectra $S_{\text {p}_0}(\lambda , \mathbf {x})$, which represent initial pressure values at a fixed spatial location $\mathbf {x}$ over all recorded wavelengths $\lambda$. The method is compared to linear unmixing approaches, as well as pO$_2$ and blood gas analysis reference measurements. Experimental results suggest that the proposed method is able to obtain sO$_2$ estimates from multispectral photoacoustic measurements in silico, in vitro, and in vivo.

## Introduction

Tissue blood oxygen saturation sO$_2$ is an indicator of the health status of a patient^1^ and can be used for numerous intra-operative applications. Furthermore, characteristic changes in local sO$_2$ are associated with some of the hallmarks of cancer^2^. State-of-the-art methods to obtain this value are limited as they are either invasive (e.g. arterial blood gas analysis^3^), lack practicability and accuracy (e.g. blood oxygen level-dependent magnetic resonance imaging^4^ or functional near infrared spectroscopy^5^), or only yield a rough global estimate from peripheral vasculature (e.g. pulse-oximetry^6^). None of these techniques yield real-time, spatially-resolved sO$_2$ estimates. Photoacoustic imaging (PAI) promises to mitigate many of these disadvantages, because it is non-invasive and provides real-time measurements of spatially-resolved sO$_2$.

Many methods that aim to achieve sO$_2$ estimation using PAI have been proposed to date^7^, including model-based inversion techniques^8–12^ and data-driven approaches^13–19^. Due to limitations in terms of applicability, repeatability, or ease-of-use, none of the advanced methods are routinely used in the field of multispectral PAI. Instead, the most commonly applied technique for estimating sO$_2$ is linear unmixing (LU)^20,21^, which assumes a linear combination of relevant chromophores (in the instance of sO$_2$: oxyhemoglobin HbO$_2$ and deoxyhemoglobin Hb) to the signal. The core assumption for LU algorithms is that the signal intensities of the reconstructed PA image *S*, which is an approximation of the underlying initial pressure distribution p$_0$, are only proportional to the optical absorption coefficients $\mu _a$ of the chromophore distribution $S(\lambda ) \approx p_0(\lambda ) \propto \mu _a(\lambda )$. This assumption does not hold in practise, because the reconstructed image is also proportional to both the Grüneisen parameter $\Gamma$, and the light fluence $\phi$: $S(\lambda ) \approx p_0(\lambda ) \propto \mu _a(\lambda ) \cdot \Gamma \cdot \phi (\lambda )$.

While $\Gamma$ is not assumed to be wavelength-dependent, the fluence $\phi (\lambda )$ is dependent on the optical tissue properties and as such the wavelength $\lambda$. Due to this interdependency, $\phi (\lambda )$ has a non-trivial and non-linear influence on the recorded multispectral signal $S(\lambda )$. As the optical absorption and scattering coefficients change with wavelength, so does the fluence, which leads to changes in PA signal. This effect is generally referred to as *spectral corruption*^8^ or *spectral coloring*^22^. Even small absorption coefficients in the background medium can lead to coloring effects, depending on the depth in the medium. Methods focusing on quantitative photoacoustic imaging have been proposed to solve the ill-posed inverse problem of estimating the optical absorption properties of tissue from the initial pressure distribution^13,16,22–24^. However, while showing great theoretical promise, these methods have not been proven to work for in vivo measurements of complex media. One reason for this is that there is a *domain gap* between simulated and real photoacoustic data. Therefore, accurately calculating blood oxygenation values from multispectral photoacoustic signals in a clinical context remains challenging^25^.

Our work introduces a data-driven approach for tackling the problem of sO$_2$ estimation by introducing the method of *learned spectral decoloring* (LSD). LSD is based on the assumption that the domain gap can be addressed by performing training on initial pressure data by using normalized pixel-wise p$_0$ spectra. If this assumption holds, it should be possible to train the algorithm using only data from PA optical forward simulations. Using a digital twin of the PA device and a synthetic representation of the target tissue, the LSD algorithm learns how spectral coloring can affect the p$_0$ spectra at different spatial locations in the tissue. In several experiments, we train the algorithm on in silico data and apply it to various in silico, in vitro, and in vivo data sets. According to the results, sO$_2$ estimates from spectrally colored data are generally feasible with LSD, which shows promising advantages compared to LU. For example, LSD consistently exhibits a higher dynamic range than sO$_2$ estimates obtained using LU techniques and enables orders of magnitude faster sO$_2$ estimation.

## Methods

**Figure 1:**
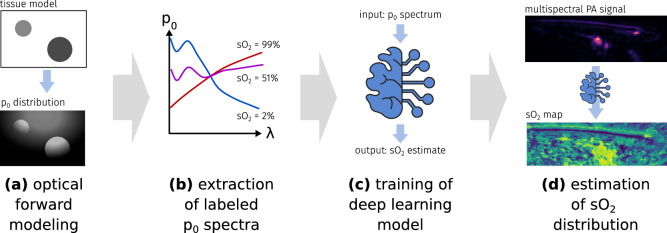
Overview of the methodology: first, numerous p$_0$ spectra are extracted from optical forward simulations (**a**). These pixel-wise spectra are retrieved from the multi-wavelength simulation by evaluating the p$_0$ intensity at a fixed pixel location as a function of wavelength (**b**). The data is then used to train a deep learning algorithm (**c**), which afterwards is able to estimate sO$_2$ values on data that comprises the same wavelengths (**d**).

This section will first introduce the principle of the *learned spectral decoloring* (LSD) method as depicted in Fig. 1, then will present the data sets, and finally will specify the implementation details.

### Concept overview

Assuming that the Grüneisen parameter $\Gamma$ is constant, $p_0$ can be expressed with the following equation: $p_0(\lambda ) \propto \mu _a(\lambda ) \cdot \phi (\lambda )$. Prior work by Tzoumas et al.^8^ has shown that it is theoretically feasible to compute pixel-wise sO$_2$ values based on $p_0$ spectra by assuming linear mixture models for both $\mu _a$ and $\phi$. Here, the optical absorption coefficient can be expressed by the weighted sum of the constituting chromophore spectra *c*: $\mu _a = \sum _k^N a_k\cdot c_k$ and the fluence can be expressed as a weighted sum of eigenspectra *e*: $\phi = \sum _k^M b_k\cdot e_k$. Both $c_k$ and $e_k$ are known *a priori*. It is assumed that the extraction of $p_0$ with sufficient wavelengths enables a reliable sO$_2$ estimation from the spectrum, as $p_0$ can only be explained by a limited amount of plausible $\mu _a$ and $\phi$ combinations. However, it is challenging to constrain a conventional minimization algorithm to yield a unique solution when used on handheld PA device geometries. In recent work of Olefir et al.^26^ it was shown that deep learning algorithms can help to mitigate these issues. One of the core assumptions of *learned spectral decoloring* (LSD) is that the optimal set of constraints for the inversion can be learned from the wavelength-dependent changes of $p_0$ using data-driven approaches. A key challenge in implementing such a method is the lack of labeled ground truth data. This is addressed by using simulated in silico training data to train a neural network that approximates a function $f_\text {LSD}$ which maps the initial pressure spectra $S_{p_0}$ to corresponding blood oxygenation saturation sO$_2$ values:

1 $$\begin{aligned} f_\text {LSD}: S_{p_0} = \left( \begin{array}{c} p_{0_{\lambda _1}}\\ \ldots \\ p_{0_{\lambda _n}} \end{array}\right) \in \mathbb {R}^n \rightarrow \text {sO}_2 \in \mathbb {R}, \end{aligned}$$

where *n* is the number of recorded wavelengths. $f_\text {LSD}$ is a neural network that is trained to compensate for different levels of spectral coloring and that learns a mapping strategy in which many differently colored p$_0$ spectra correspond to the same sO$_2$ value. Due to an inherent lack of ground truth sO$_2$ values for experimental p$_0$ measurements, the method is trained on simulated data sets that can be optimized for the specific applications and wavelengths. Many samples of differently colored spectra are obtained from the same in silico sample by extracting single-pixel spectra from multiple spatial locations. For example, the influence of the gradual absorption of energy by water is expressed to a greater extent for deep samples, as there has been a greater amount of interaction between the light and the chromophore. This leads to different $p_0$ spectra for the same set of optical parameters. A visual representation of the spectra extraction from simulated p$_0$ data is shown in Fig. 1.

During training, the algorithm is given tuples $\left( S_{p_0}, \text {sO}_2\right)$, with $S_{p_0} \in \mathbb {R}^n$ and $\text {sO}_2 \in \mathbb {R}$. Each spectrum $S_{p_0}$ is normalized such that all vector components sum to one $(\sum _{i=1}^n S_{p_{0_{\lambda _i}}} = 1)$.

The amplitude information of the recorded spectra is crucial for quantitative photoacoustic imaging which aims to obtain absolute concentrations of chromophores in tissue. However, by sacrificing the amplitude information, we also eliminate the need to calibrate the in silico training data to the acquisition device and the specific target domain. This drawback is tolerable, as we are only interested in the relative ratios of HbO$_2$ and Hb. Furthermore, it constitutes an important step towards bridging the *domain gap* and thereby enables the LSD method to be applied to real data with unknown calibration of the PA measurement device. Real data needs to be reconstructed from raw time series pressure data in order to form a spatial image of signal amplitudes. A reconstruction algorithm can introduce artifacts to the image, for example, due to limited view geometries^27^. However, in this work, the biases introduced by the reconstruction are assumed to be independent of the wavelength, allowing the algorithm to be directly trained on $p_0$ data without the need to simulate the acoustic forward model as well.

### Data sets

**Figure 2:**
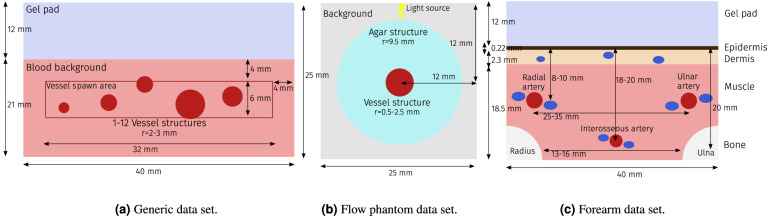
Schematic representation of the three in silico data sets. (**a**) shows the generic data set, (**b**) depicts the in silico flow phantom data set, and (**c**) visualizes the structures of the forearm data set. The vascular structures are simulated as tubes. The dark blue structures specifically correspond to veins.(**a**, **c**) were simulated with a digital twin of a custom PAI device based on the DiPhAs imaging system (Gröhl et al.^28^), whereas (**b**) was simulated with a pencil beam as the illumination source.

Several data sets were used in this work for training and validation. These validation data comprised (a) an in silico data set, (b) in vitro data set from a blood flow phantom, and in vivo data sets from (c) an open porcine brain and (d) forearms of healthy human volunteers. The purpose of the in silico data sets was to evaluate the sO$_2$ estimation accuracy that could potentially be achieved by the LSD method; the in vitro data set was used to investigate if the method is capable of recovering the entire value range when the sO$_2$ is chemically decreased from 100% to 0% in a controlled manner and the in vivo data sets were used to demonstrate that the method is capable of obtaining plausible values in real data derived from complex media.

- **Synthetic data.** A total of three simulated data sets were generated to train the models and test the method in different scenarios (Fig. 2). All of these had different a priori assumptions for the underlying optical tissue properties.
  1. The *generic data set* (Fig. 2a). This data set contained a generic tissue representation without skin-specific chromophores such as melanin and was used for estimations of the open brain data. It contained randomly distributed vessel structures with 100% blood volume fraction, a homogeneous background medium with 0.5% blood volume fraction, and a scattering coefficient of 10 cm$^ {-1}$. All structures were initialized with the same random blood oxygenation levels that were drawn from a uniform distribution from 0% to 100% oxygenation. The phantoms were simulated with 26 wavelengths, equidistant from 700 nm to 950 nm, using a multi-threaded adaptation of the Monte Carlo framework mcxyz^29^ with $10^7$ photons for each simulation.
  2. The *flow phantom data set* (Fig. 2b). This data set was designed to resemble the geometric setup of the oxygenation flow phantom as presented by Gehrung et al.^30^. Following the specifications of the agar phantom, the structure was assumed to have a reduced scattering coefficient $\mu _s'$ of 5 cm$^{-1}$. For increased variability, we randomly changed the water content between 50 and 100%. The agar structure contains a single tubular structure containing blood with a haemoglobin concentration of 150g/L and an oxygenation level uniformly randomized between 0 and 100%. In order to reduce the influence of discretization artifacts and to increase the effective number of samples, the radius of the tube was uniformly randomized between 0.5 and 2.5 mm for each simulation. The phantoms were simulated with the same wavelengths used for data acquisition: {660, 664, 680, 684, 694, 700, 708, 715, 730, 735, 760, 770, 775, 779, 800, 850, 950} nm. The MCX simulation framework^31,32^ was used to simulate this data set due to its fast computational speed with $10^7$ photons for each simulation.
  3. The *forearm model data set* (Fig. 2c). The forearm data set was designed to mimic all structures in the human forearm, including the chromophore melanin that is present in the epidermis. The synthetic forearm phantoms were also simulated with 26 wavelengths, equidistant from 700 to 950 nm, using the MCX simulation framework with $10^7$ photons for each simulation. The optical properties of the different structures were modeled as reviewed by Jacques^33^ and a constant anisotropy of $g=0.9$ was assumed. Table 1 shows the assumed physiological ranges for different parameters in the respective structures.

**Table 1:** Assumed property ranges and chromophore abundances for the different tissue types.

Tissue type	Blood volume [%]	Oxygenation [%]	Melanin [%]	Water [%]
Gel Pad	0	–	0	0
Epidermis	0	–	2.2 ± 1^34^	0
Dermis	1	80 ± 10	0	58^35^
Muscle	1	80 ± 10	0	68^35^
Vessel	100	0–100	0	0
Artery	100	0.95 ± 5^36^	0	0
Vein	100	70 ± 10^37^	0	0
Bone	0	–	0	19 ± 1^38^

For each instance of a forearm phantom was created, random values for ranges *X*–*Y* were drawn from a uniform distribution and values for ranges $X \pm Y$ were drawn from a Gaussian normal distribution. The given values refer to the volume fraction of the chromophore. Here, whole blood was assumed to have a hemoglobin concentration of 150 g/L^33^.
  For all simulated data sets, spectra were extracted from a region of interest (ROI), defined as vessel structures where the signal at the isosbestic point of 800 nm was higher than a noise equivalent threshold (determined by calculating the pixel-wise contrast-to-noise ratio (CNR) and setting a threshold of CNR $\ge 2$). This was done because hardware limitations in overall sensitivity and acoustic frequency responses make it impossible to extract any meaningful information hidden deep in a blood vessel in in vivo images.
- **Blood flow phantom data.** This data set consisted of three measurements of a blood flow phantom setup, including reference blood oxygenation measurements provided by partial oxygen pressure (pO$_2$) needle probes. It contains measurements of two human blood samples and a rat blood sample. A diagram of the measurement setup and a detailed description of the data acquisition process can be found in Hacker et al.^39^. The data was measured at the University of Cambridge using an *MSOT inVision 256-TF* imaging system (iThera Medical GmbH, Munich, Germany). The blood samples were first chemically oxygenated and then chemically deoxygenated during the measurement process, theoretically going from 100% blood oxygenation to 0% blood oxygenation over the measurement time, with continuous reference measurements being taken by pO$_2$ needle probes. These pO$_2$ measurements were translated into sO$_2$ estimates using the Severinghaus equation^40,41^. For the evaluation of the method on this data set, ten consecutive frames of the same wavelength were averaged to account for laser pulse energy fluctuations. The tube structure was automatically segmented by only taking pixels in which the signal at 800 nm was greater than $2 \times 10^4$ MSOT signal units into account. This threshold was chosen to yield a good fit of the vessel structure for each of the data sets. This step was necessary, as the tubular structure was subject to slight movements over the imaging duration, and as such, it was not feasile using a constant manually-segmented ROI for all of the images.
- **Porcine brain data.** This data set consists of a multispectral image series that was taken from a previous animal experiment in which a porcine brain was imaged during open brain surgery^42^. The used images were acquired as part of a pilot study to see the hemodynamic responses of the brain during spreading depolarization. The specific data used for this study corresponded to a baseline measurement done to establish the capability of PAI to distinguish different hemodynamic states which were induced by using different levels of respiratory oxygen. During the entire length of the imaging procedure, 38 minutes, the animal was supplied with different levels of respiratory oxygen (rO$_2$) mixed in the mechanical ventilation air flow, to induce changes in the hemodynamics of the brain. Specifically, the rO$_2$ was set to these values during the experiment: 35% from minute 0 to 5 (baseline), 21% from minute 5 to 10 (normoxia), 0% from minute 10 to 16 (anoxia), started recovery with 21% from minute 16 to 21 (normoxia), and 100% from minute 21 to 26 (hyperoxia). Finally, the rO$_2$ was again set to the baseline (35%) for the remainder of the experiment. Towards the end of each interval, we took arterial blood gas measurements as a reference. In the original study, we used three month old female German Landrace swines weighing 30–35 kg with a sample size of N = 3. In this evaluation, we used the experiment that had the most reliable blood gas measurements as assessed by a clinician and analysed the blood oxygenation over time using linear unmixing and learned spectral decoloring on the photoacoustic measurements. The images were recorded at the same wavelengths as in the training data set (700–950 nm at intervals of 10 nm). They were normalized by the recorded laser energy and reconstructed with the delay-and-sum algorithm using a hamming window and were recorded using a custom PAI device based on the DiPhAs imaging system (Kirchner et al.^43^). Further details on the experimental design and the general image acquisition can be found in previous publications of the experiments^42,44^.
- ** Human forearm data.** This data set consists of multispectral images of the left and right forearms of three healthy human volunteers. The images were taken at three distinct positions a distance of approximately 2, 4, and 6 cm from the radiocarpal joint, leading to a total of 18 image sequences. The person operating the handheld PAI device attempted to capture either the *arteria radialis* or the *arteria ulnaris* in the imaging plane. These vessels could be identified by their pulsating motion which is induced by the heartbeat. The data sets were recorded using the MSOT Acuity Echo PAI device (iThera Medical GmbH, Munich, Germany). The images were taken at a wavelength range from 700–950 nm in intervals of 10 nm for at least 30 s. The ten subsequent multispectral sequences with the least amount of movement were averaged to account for laser intensity fluctuations and to increase the robustness against motion artifacts. Finally, the images were scaled to a size of 256 $\times$ 128 pixels. The vessel structures were manually annotated using the corresponding PA and US images. For the final segmentation masks only pixels were considered that had a CNR $\ge 2$ and vessels containing less than 20 pixels were excluded to ensure a relevant sample size for the sO$_2$ estimates.

### Deep learning models

**Figure 3:**
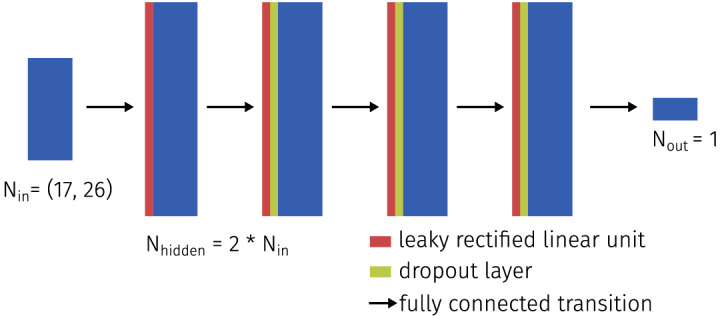
Visualization of the network architecture used for this work. The hidden layer has a size of twice the input layer. The blue color represents a layer of the network, the black arrows correspond to a fully connected transition, where every neuron of the previous layer is connected to the next. Red and green represent leaky rectified linear units and dropout layers, respectively.

For each of the training data sets, a separate fully connected feed-forward neural network was trained to account for domain-specific differences. The feed-forward architecture was chosen because we decided to use a well-understood baseline method for this initial study and a single-pixel approach does not warrant the use of convolutional neural networks. The number of input features was set to the number of wavelengths in the respective multispectral sequence (26 for the forearm and generic data set and 17 for the flow phantom data set), the model contained four hidden layers and the size of these hidden layers was set to be twice the size of the input vector (see Fig. 3).

The models were trained for 100 epochs, where one epoch contained 500 batches of size $10^4$. The initial learning rate was set to $10^{-2}$ and was updated every two epochs to $\text {new}_\text {lr} = 10^{-2} \times 0.9 ^{(\text {epoch}/2)}$. After each leaky rectified linear unit, we applied a dropout of 20% to prevent the network from overfitting. Tracking of the validation losses over the number of epochs showed that the validation loss did not significantly decrease after as little as ten epochs using this training scheme. For each synthetic data set, a model was trained on 75% of the available data, 5% of the data were used for validation and the final 20% of the data was used as a held-out test set. The reported hyperparameters were optimized based on the performance of the method on the validation set. The test set was only evaluated once, at the end of the training process, to obtain the final results.

### Linear unmixing

As the LU technique constitutes the state-of-the-art method in functional parameter estimation for photoacoustic imaging, it was used as a reference method to compare the proposed LSD method to. It was performed using literature absorption spectra of pure Hb and HbO$_2$ as reviewed by Jacques^33^. The unmixing method was implemented in Python 3.7., using the *minimize* function of the *scipy* python package that implements the SLSQP (Sequential Least SQares Programming) algorithm for finding the best fit. The unmixing was done exclusively for Hb and HbO$_2$, using initial values of 0.5.

### Ethical approval

The healthy human volunteer experiments were carried out in accordance with relevant guidelines and regulations and were approved by the ethics committee of the medical faculty of Heidelberg University under reference number S-451/2020. The study is registered with the German Clinical Trials Register under reference number DRKS00023205. All porcine experiments were carried out in accordance with relevant guidelines and regulations, including the ARRIVE guidelines, and protocols were approved by the institutional animal care and use committee in Karlsruhe, Baden-Wuerttemberg, Germany (Protocol No. 35-9185.81/G-174/16).

### Informed consent

Informed consent was obtained from all volunteers.

## Results

**Figure 4:**
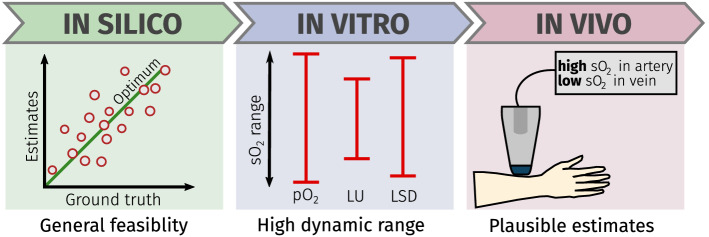
Stylized summary of the key findings of the experiments. The in silico experiments demonstrated the general feasibility of the LSD method, the in vitro experiments revealed the large dynamic range of the LSD estimates, and the in vivo experiments showed that the method yields more plausible estimates than LU even in complex situations.

This section presents the results of the LSD method on the simulated in silico data sets, on the in vitro flow phantom data sets, and on the in vivo porcine brain and human forearm data. A separate LSD regressor was trained for each of the respective in silico training data sets. The key findings of the experiments are summarized in Fig. 4.

### In silico results

**Figure 5:**
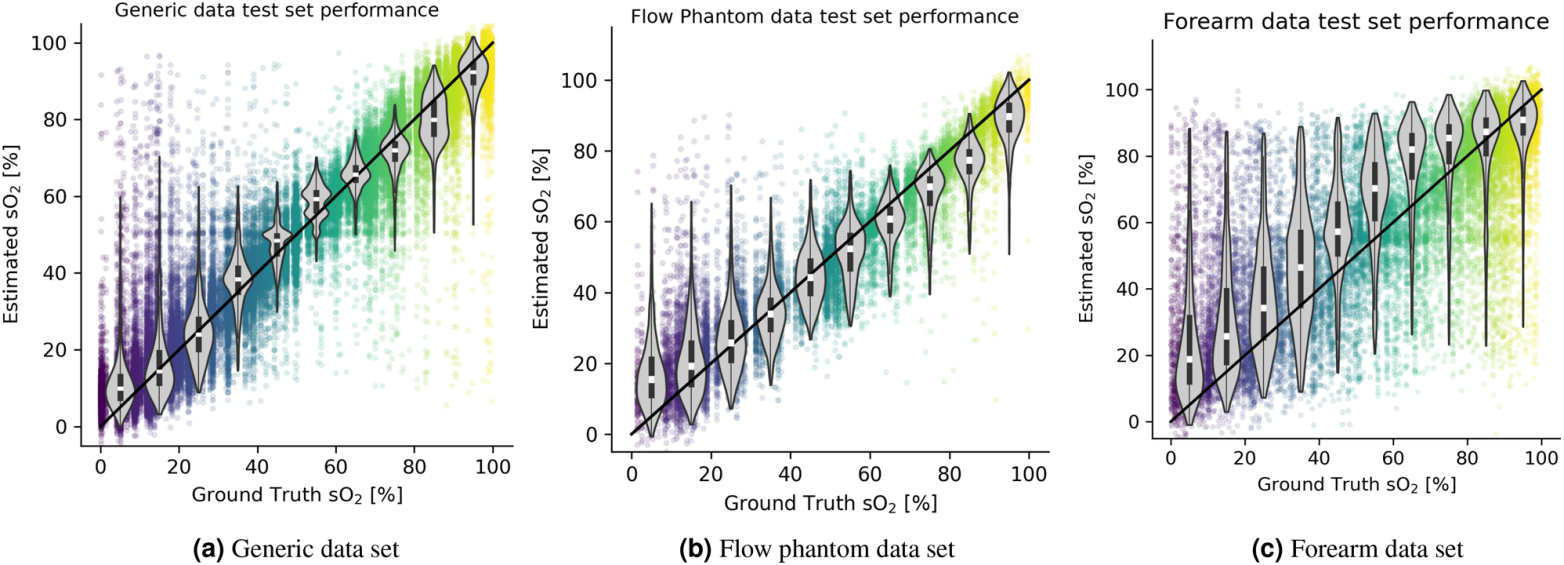
In silico estimation results for the generic data set (**a**), the flow phantom data set (**b**), and the forearm data set (**c**). The scatter plot is colored with the ground truth oxygenation value. The violin plots show the estimated sO$_2$ for the ground truth sO$_2$ intervals in increments of 10%. As such, in addition to the scatter plot, there is one violin plot for all ground truth sO$_2$ values in equidistant steps of 10%.

Figure 5 illustrates the performance of the respective deep learning model when tasked with predicting sO$_2$ values for the test set from the generic data set, the flow phantom data set, and the forearm data set. Here, the relative sO$_2$ estimation error is reported, which was calculated using the equation $e_{\text {sO}_2} = |\text {sO}_2^\text {EST} - \text {sO}_2^\text {GT}| / \text {sO}_2^\text {GT}$, with $\text {sO}_2^\text {EST}$ being the estimated oxygen saturation and $\text {sO}_2^\text {GT}$ being the ground truth oxygen saturation.

The median relative sO$_2$ estimation error for the model trained and tested on the generic tissue model data set was 6.1%, with an interquartile range (IQR) of (2.4%, 18.7%). On the flow phantom data set, the LSD method achieved a median relative estimation error of 9.9%, with an IQR of (3.6%, 28.5%). The largest error produced by the LSD method was found in the in silico forearm data set with a relative median quantification error of 15.0%, and an IQR of (5.3%, 45.4%).

The median *absolute* sO$_2$ quantification error on the test sets was well below 10 percentage points for all data sets. The model that was trained and tested on the forearm data set achieved 7.9 percentage points median absolute sO$_2$ estimation error with an IQR of (3.5, 17.1) percentage points.

### In vitro results

**Figure 6:**
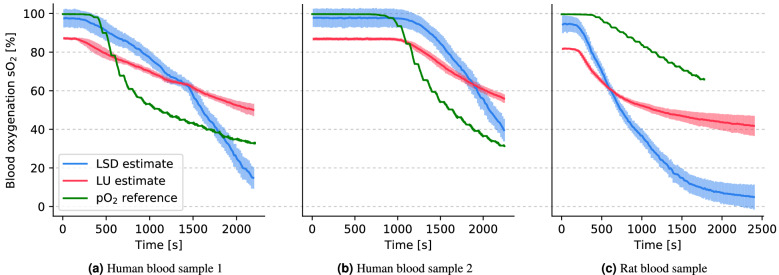
The mean oxygenation estimation results from three different measurement methods, shown over time on three different blood samples: (1) LSD in blue, (2) LU in red, and (3) pO$_2$ reference measurement in green. The standard deviation of the estimations within the ROI for LU and LSD unmixing is shown around the mean estimate in the corresponding color. The graphs are shown for human blood samples (**a**) and (**b**) and for a rat blood sample (**c**).

For a comparative analysis of the flow phantom data, three techniques of sO$_2$ estimation are shown in Fig. 6: spectral unmixing using LU, spectral unmixing using the proposed LSD approach, and pO$_2$ probe reference measurements. The mean and standard deviation of the estimates for both the LU and LSD approach are shown on the graphs.

All three of these measurements showed a monotonous decrease in the blood oxygenation level over the time frame of the experiment. The pO$_2$ reference measurements yielded a value range from $\approx$ 100% to 5% blood oxygenation. In contrast, LU exhibited a dynamic range of $\approx$ 85% to 40%, whereas LSD results exhibited a dynamic range of $\approx$ 95% to 5% on all three data sets. It should be noted that the pO$_2$ references could not be perfectly aligned due to constraints of the equipment and procedure. The temporal calibration of the human blood sample (b) was manually adjusted by 1000 s, as the curves did not seem to match initially. Furthermore, the pO$_2$ reference was stopped prematurely in the rat blood data set.

**Figure 7:**
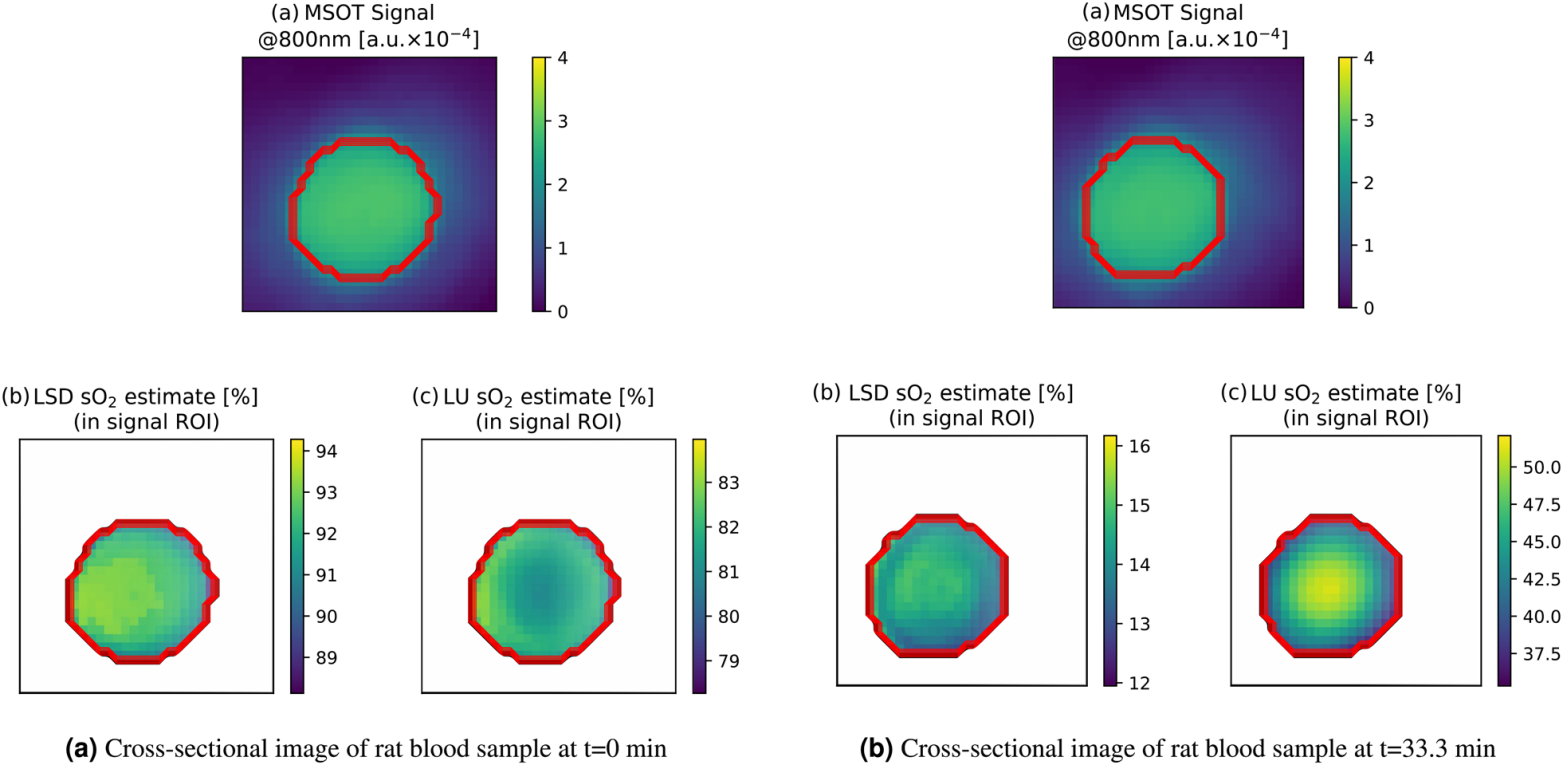
Visualization of a cross-sectional view through the flow phantom rat blood data. The left plot (**a**) shows the spatial distribution of sO$_2$ estimates in the beginning of the experiment (t = 0 min) and the right plot (**b**) towards the end of the experiment (t = 33.3 min). The rim-core differences in the sO$_2$ estimates are more pronounced in LU when compared to LSD.

In addition, Fig. 7 shows the depth-dependent behavior of the unmixing result for two time-points in the measurement. In both cases one can clearly see, that the rim-core differences in the sO$_2$ estimates are more pronounced in LU when compared to LSD. The LSD estimates are more homogeneous throughout the imaging cross section.

### In vivo results

**Figure 8:**
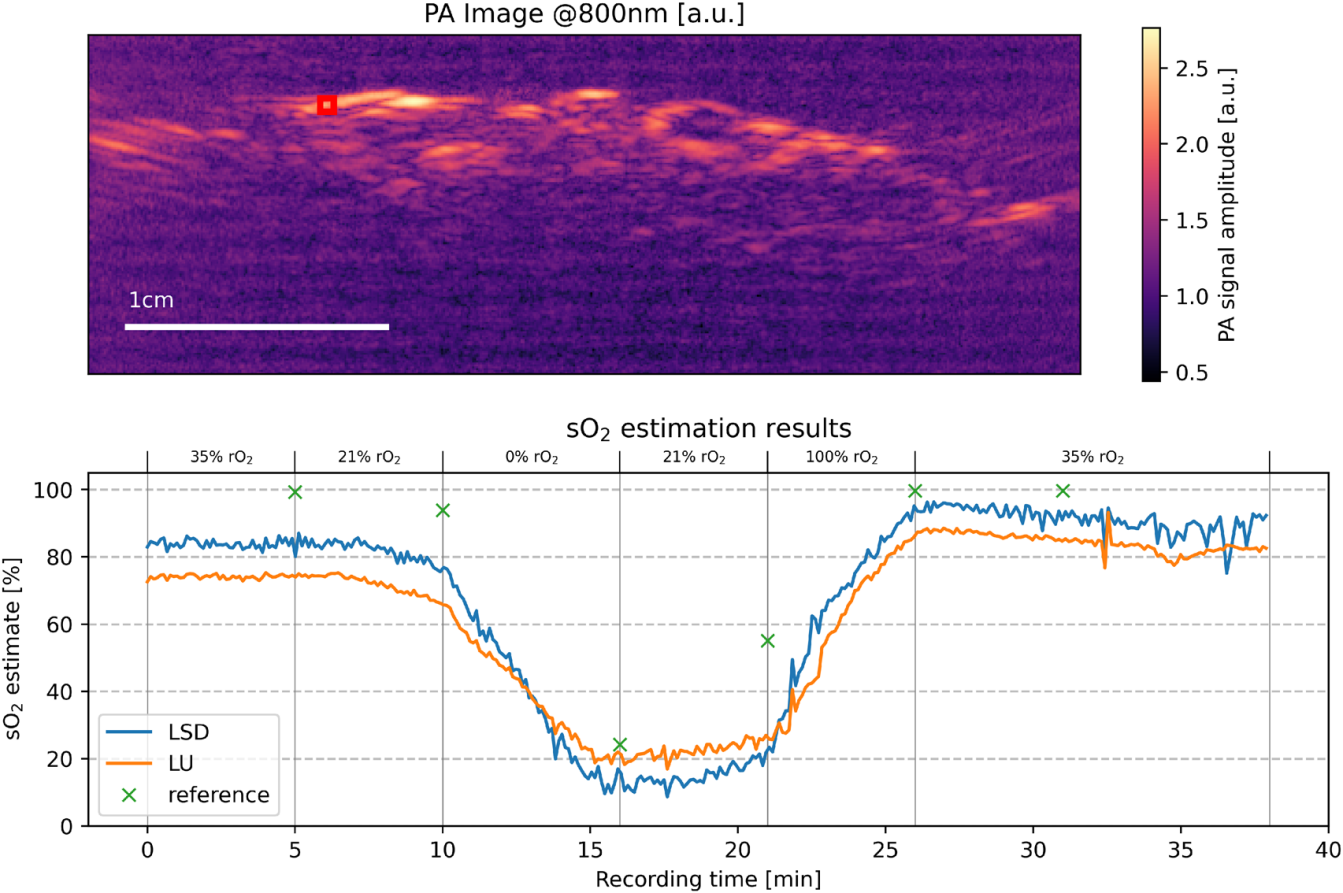
The results of LSD in vivo on an open porcine brain with a deep learning model trained on the generic tissue data set. The LSD results are compared to the LU results. The red rectangle shows an ROI which the LSD (blue) and LU (orange) results were computed on. The green crosses mark the time points and values of reference arterial blood gas analysis (BGA) measurements.

In the porcine brain image series, LSD was observed to increase the dynamic range of the predictions while maintaining the same tendency (high values were mapped to higher values and low values were mapped to lower values). Median oxygenation values were computed and tracked over time in a manually placed ROI in Fig. 8. In addition to the comparison of LSD and LU, arterial blood gas analysis measurements were taken and are shown in the figure as well.

**Figure 9:**
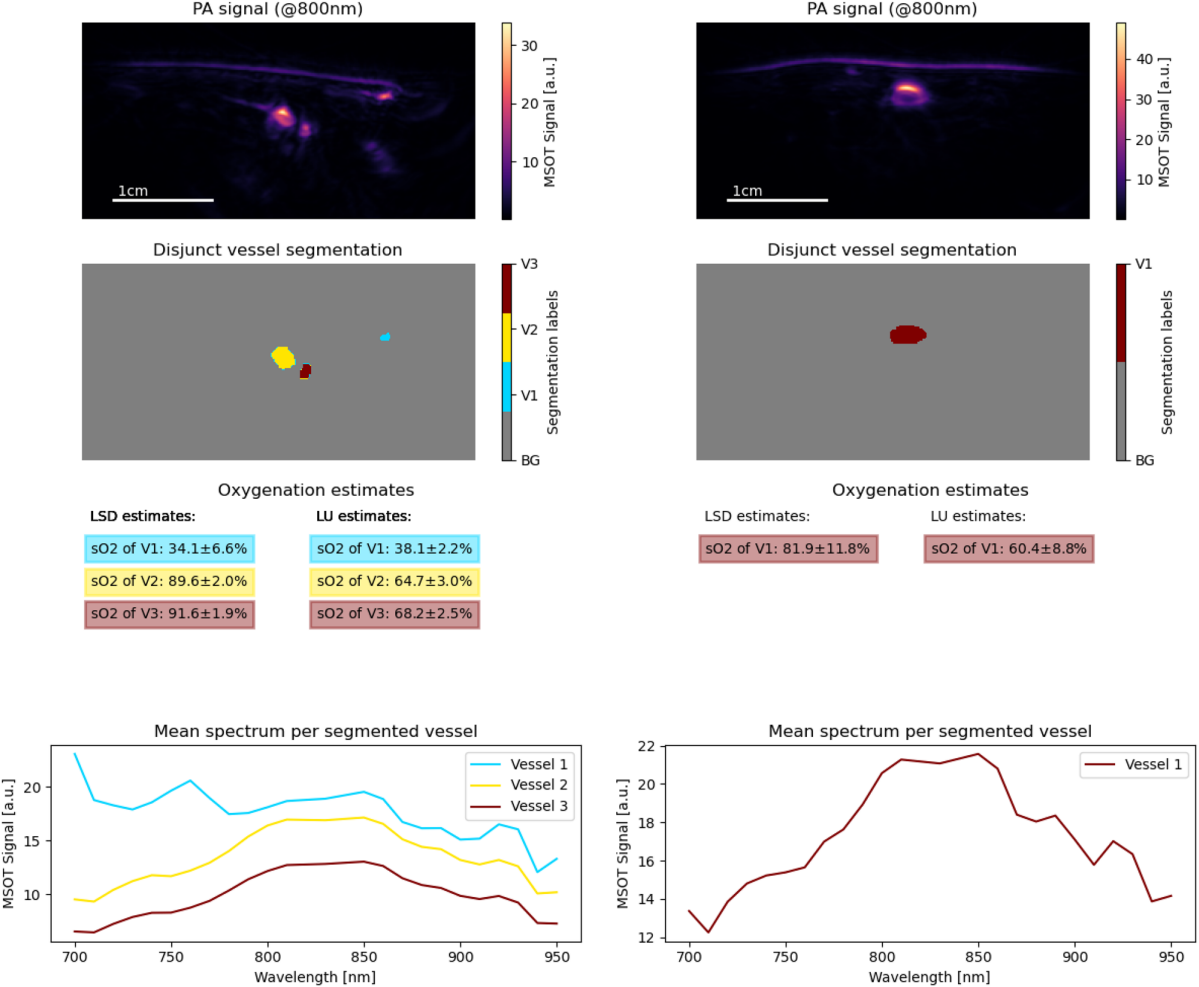
The results of LSD in vivo on the forearms of two healthy human volunteers. The LSD model was trained on the in silico forearm data set. The results were compared to the results for data analyzed with LU. The top row shows the PA signal at 800 nm, the second row shows the segmentation masks of the imaged vessels, the third row compares the LU and the LSD sO$_2$ estimates, and the last row shows the mean MSOT signal spectrum for each of these vessels.

Figure 9 shows the results of the method on the in vivo forearm data set on two example images. For completeness, the results on all 18 image slices are available in the Supplemental Material S1. The LSD estimates were obtained from a deep learning model trained on the synthetic forearm data set. The results were compared to data analyzed with LU. On the left image, the arterial vessel structures are estimated to have a blood oxygenation levels of 64.7% with LU and 89.6% with LSD (yellow) and 68.2% with LU and 91.6% with LSD (red). On the right image, LSD estimates a blood oxygenation level of 81.9% for the artery (red), while LU yields 60.4%. The mean spectra of these ROIs are plotted in the figure.

## Discussion

This paper introduces the machine learning-based method *LSD*, which is able to account for the spectral coloring effects in multispectral photoacoustic imaging when estimating the blood oxygen saturation of tissue. Other recently published deep learning-based methods have only reported results on simulated data^14–17^, or preliminary results on simple phantom setups^18,19^. This shortcoming in the field may be attributed to the *domain gap* between simulated data and real measurements. LSD is based on the assumption that the consideration of spatial relations in PA measurements in simulations amplifies the *domain gap* between simulation and reality. Therefore, it is trained on single-pixel p$_0$ spectra at different spatial locations within tissue. We demonstrated that this method is able to predict plausible blood oxygenation levels in vivo and has distinct advantages compared to linear spectral unmixing as shown in both in vitro and in vivo data sets.

In vivo measurements of the forearms of healthy human volunteers suggest that LSD is capable of yielding physiologically plausible sO$_2$ measurements. In line with the expected literature values for physiological value ranges of blood oxygenation, highly oxygen saturated blood is systematically estimated to have higher sO$_2$ with LSD than LU, while poorly oxygenated blood is systematically estimated to have lower sO$_2$ with LSD than LU, bringing the estimations closer to the anticipated ground truth. This conclusion is supported by the performance of the LSD method on in vitro flow phantom data. Here, LSD exhibits a higher dynamic range of the sO$_2$ estimates than LU while showing a monotonous decrease in sO$_2$ that could be confirmed with pO$_2$ reference measurements. While the steady decrease of the sO$_2$ measurements is apparent for all three approaches, the pO$_2$ measurements decrease at different rates compared to both LSD and LU. One reason for this mismatch might be the empirical nature of the Severinghaus equation, which was used for translating the measurements into sO$_2$ values, along with an unknown pH value and a temperature mismatch. Moreover, as the pO$_2$ probe could not be placed inside the PAI system, PA and pO$_2$ measurements were made at different positions along the blood tubing leading to further misalignments. In future work, this might be remedied by adding blood gas analyzer measurements as a reference, by using a more physiological blood gas oxygenation process instead of chemical oxygenation, or by creating a ground truth reference using a phantom setup in which, a priori, the exact absolute concentrations of the mixed chromophores are known. Nevertheless, the LSD approach shows great promise on this data set, through the expanded dynamic range of the response and by significantly reducing the rim-core effect that is a sign of spectral coloring in depth. An undisputed advantage of machine learning methods is their fast inference speed after training. For a 256 $\times$ 128 pixels image with 26 wavelengths, the inference time was measured to be as low as 211 ± 9 ms using a central processing unit (CPU) (AMD Ryzen 5 1600 Six-Core Processor) and 2.4 ± 0.4 ms using a graphics processing unit (GPU) (Nvidia GTX 1080ti, 11 GB). As such, the real-time capability of this method would mostly be impeded by the hardware constraints and not by the method. For example, slow sO$_2$ estimation times can be expected when using many wavelengths and averaging steps on a system with a low pulse repetition rate.

The LSD method is capable of performing estimations in realistic settings, despite being trained on simulated data, demonstrating the ability of the approach to bridge the domain gap between real and simulated images. In fact, to our knowledge, no prior work has successfully applied convolutional neural network-based approaches to sO$_2$ estimation on entire images of initial pressure in vivo ^45^. On the other hand, since single-pixel spectra can be highly ambiguous when considering a too large solution space, thus we strike a careful balance between generalizability and applicability of the method by designing a suitable data set for a specific application. In addition to this trade-off, another severe limitation of the method is that the inversion model is wavelength-dependent, which means that a trained model can only estimate sO$_2$ values for a specific set of pre-determined wavelengths. Furthermore, while the variation in the target structures included in the data sets increased, so did the level of inaccuracy of the in silico sO$_2$ inversion results. Only in the forearm data set the background and the vessel structures were allowed to take different oxygenation values. This can increase the difficulty for inversion because a source of ambiguity is added to the problem. In order to obtain more accurate results in these cases, techniques for spatial regularization could be employed in future to counteract the ambiguity of the inversion problem. We trained the LSD models directly on p$_0$ spectra and thus assume that the acoustic forward process is independent of the incident wavelength and has no major influence on the spectral behavior. Considering the impact that reconstruction algorithm-specific artifacts can have on the spectra it may be beneficial to incorporate acoustic forward modelling and image reconstruction into the simulation pipeline in the future.

In summary, LSD provides encouraging advantages over conventional LU techniques for sO$_2$ estimation: the expanded dynamic range of the response, the significantly reduction of rim-core effects that are a sign of spectral coloring in depth, as well as fast inference times. Thus, the method comprises a promising framework for more accurate estimations of PAI biomarkers in the future.

## Supplementary Information

Supplementary Information 1.

## Data Availability

Supplemental code and data is available on zenodo: 10.5281/zenodo.4304359
