# Supplementary material for: Learned spectral decoloring enables photoacoustic oximetry
Source: Sci Rep. 2021 Mar 22;11:6565. doi: 10.1038/s41598-021-83405-8 (PMC7985523; doi:10.1038/s41598-021-83405-8)
Supplement: Supplementary file 1 — Supplementary Information 1. [file 41598_2021_83405_MOESM1_ESM.pdf]

Supplemental material: Learned spectral decoloring enables photoacoustic oximetry

Janek Gröhl[1,2,*], Thomas Kirchner[3], Tim J. Adler[1,4], Lina Hacker[7], Niklas Holzwarth[1,5], Adrián Hernández-Aguilera[6], Mildred A. Herrera[6], Edgar Santos[6], Sarah E. Bohndiek[7,8], and Lena Maier-Hein[1,2,*]

[1]German Cancer Research Center, Computer Assisted Medical Interventions, Heidelberg, Germany
[2]Heidelberg University, Medical Faculty, Heidelberg, Germany
[3]Bern University, Institute of Applied Physics, Biomedical Photonics, Bern, Switzerland
[4]Heidelberg University, Faculty of Mathematics and Computer Science, Heidelberg, Germany
[5]Heidelberg University, Faculty of Physics and Astronomy, Heidelberg, Germany
[6]Department of Neurosurgery, Heidelberg University Hospital, Heidelberg, Germany
[7]Department of Physics, University of Cambridge, JJ Thomson Avenue, Cambridge, CB3 0HE, UK
[8]Cancer Research UK Cambridge Institute, University of Cambridge, Robinson Way, Cambridge, CB2 0RE, UK

ABSTRACT

In this supplemental material we show all result images of applying the LSD method to the human forearm data.

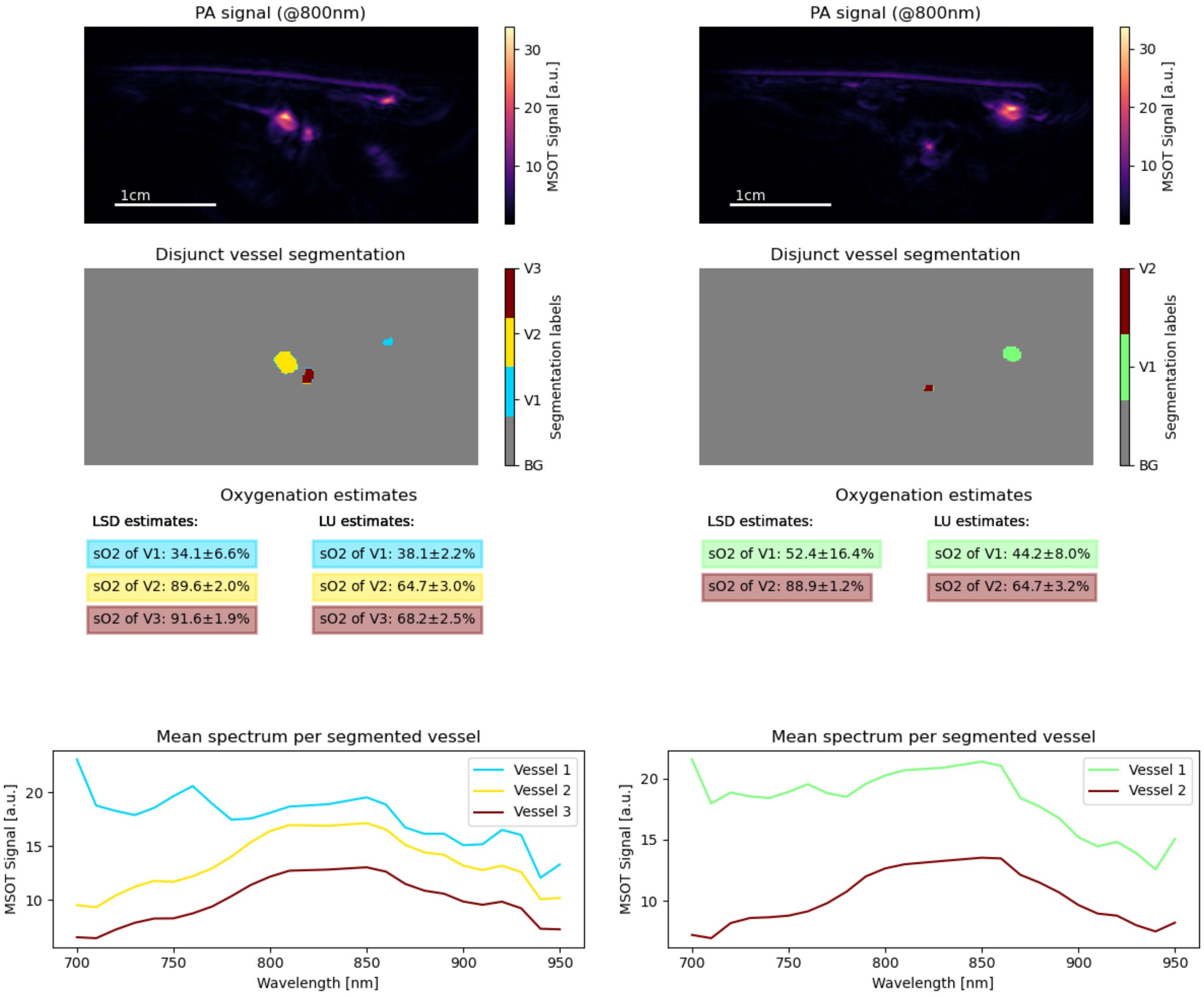

Figure 1. Result images from the human forearm data set.

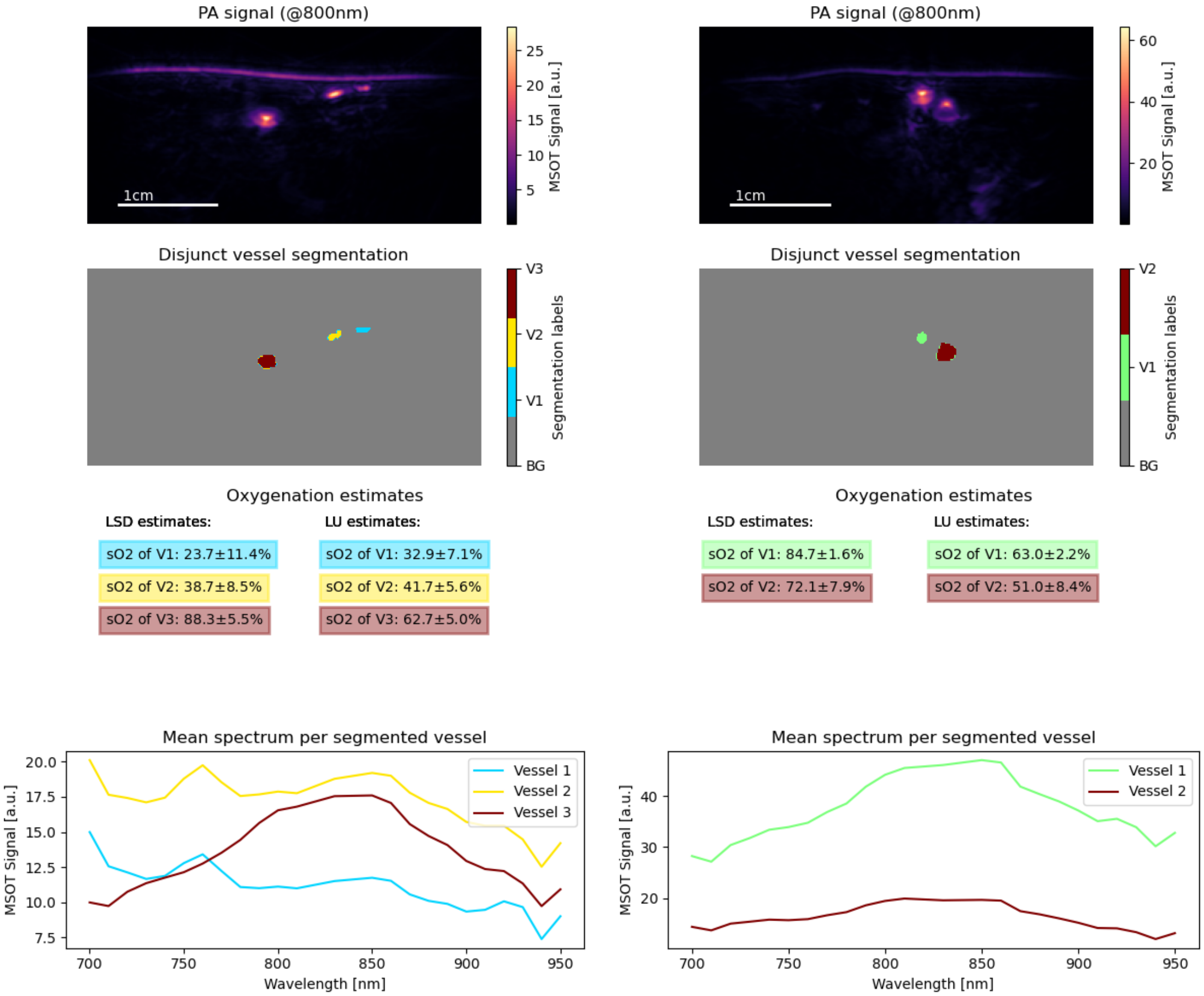

Figure 2. Result images from the human forearm data set.

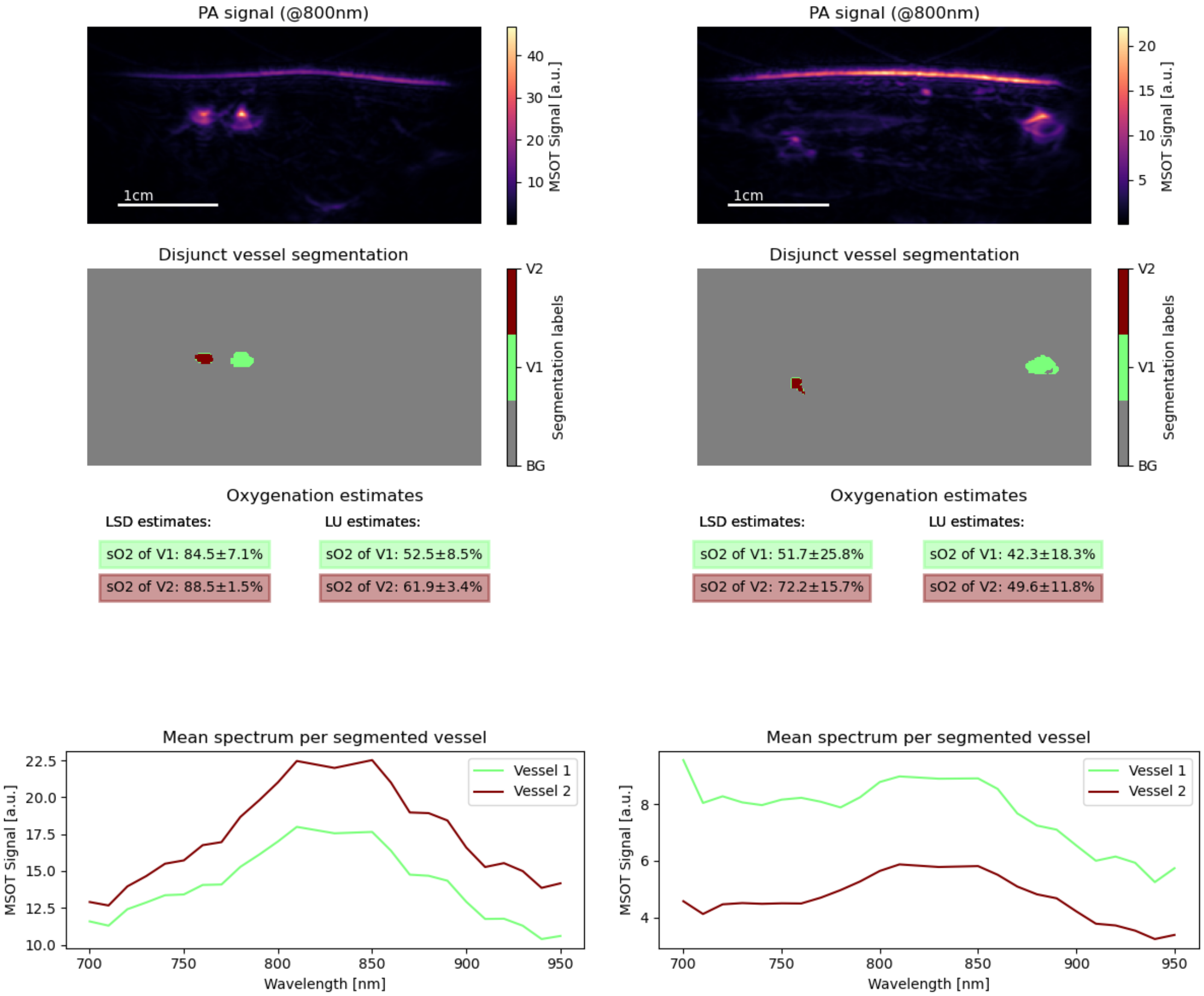

Figure 3. Result images from the human forearm data set.

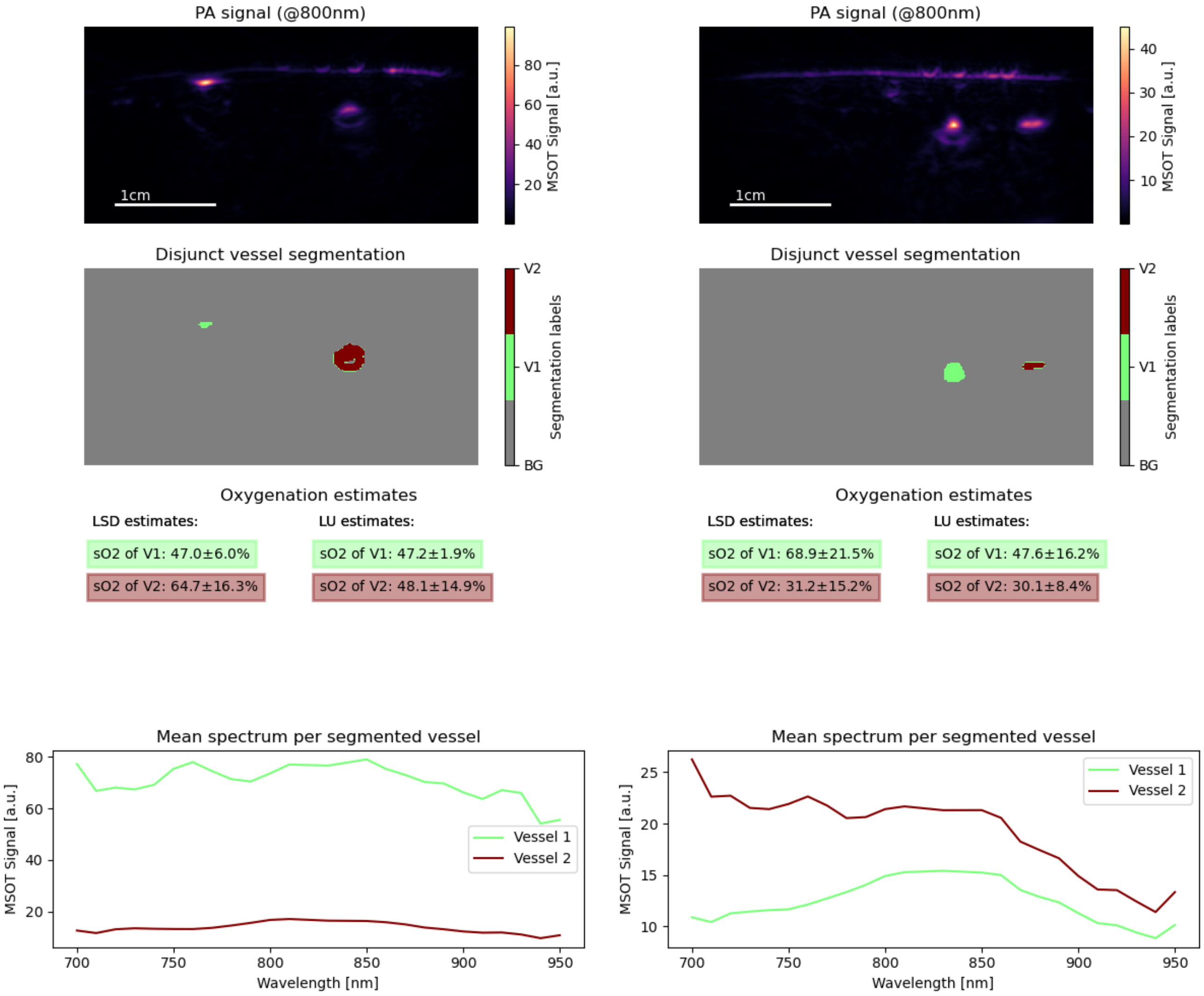

Figure 4. Result images from the human forearm data set.

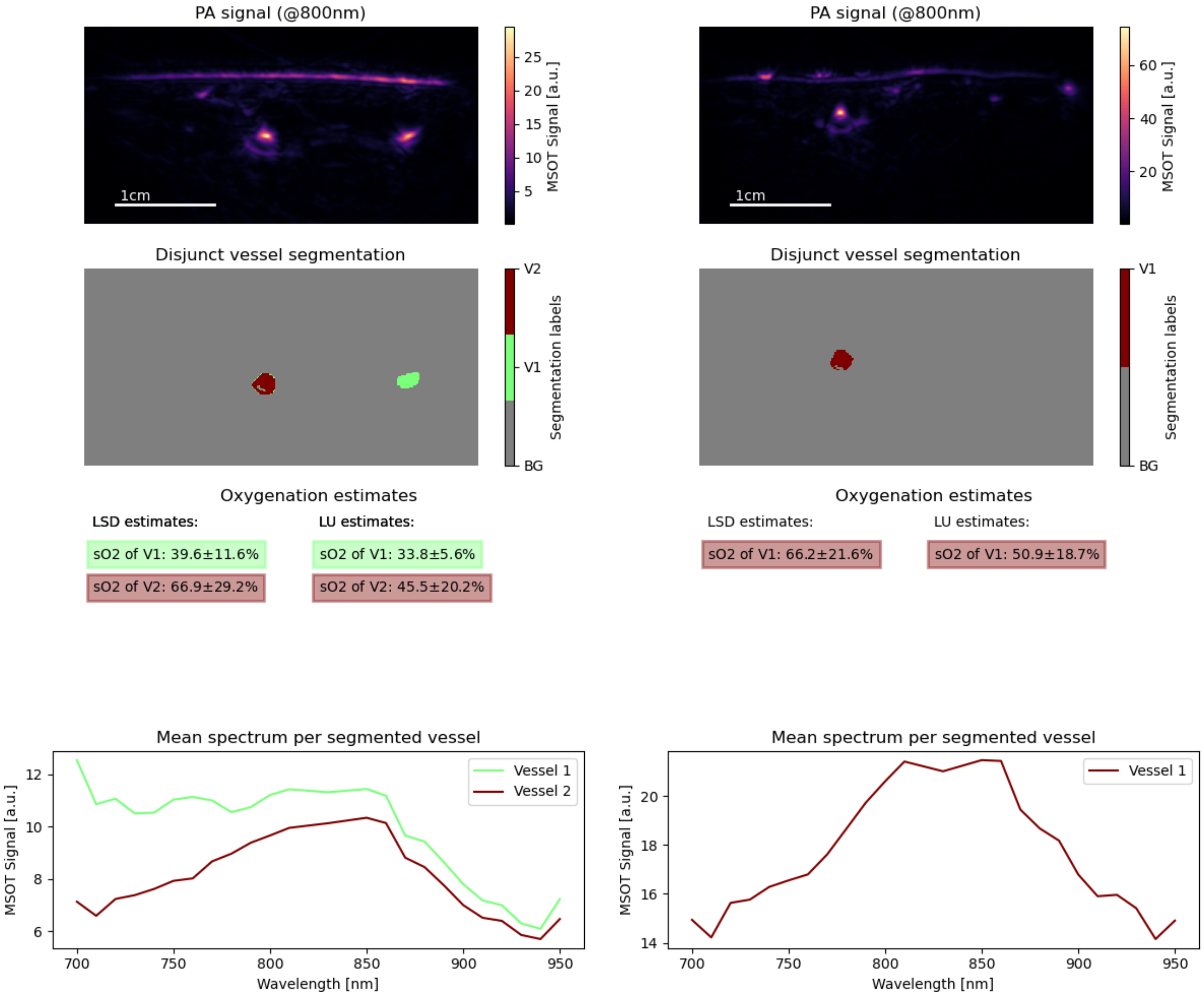

Figure 5. Result images from the human forearm data set.

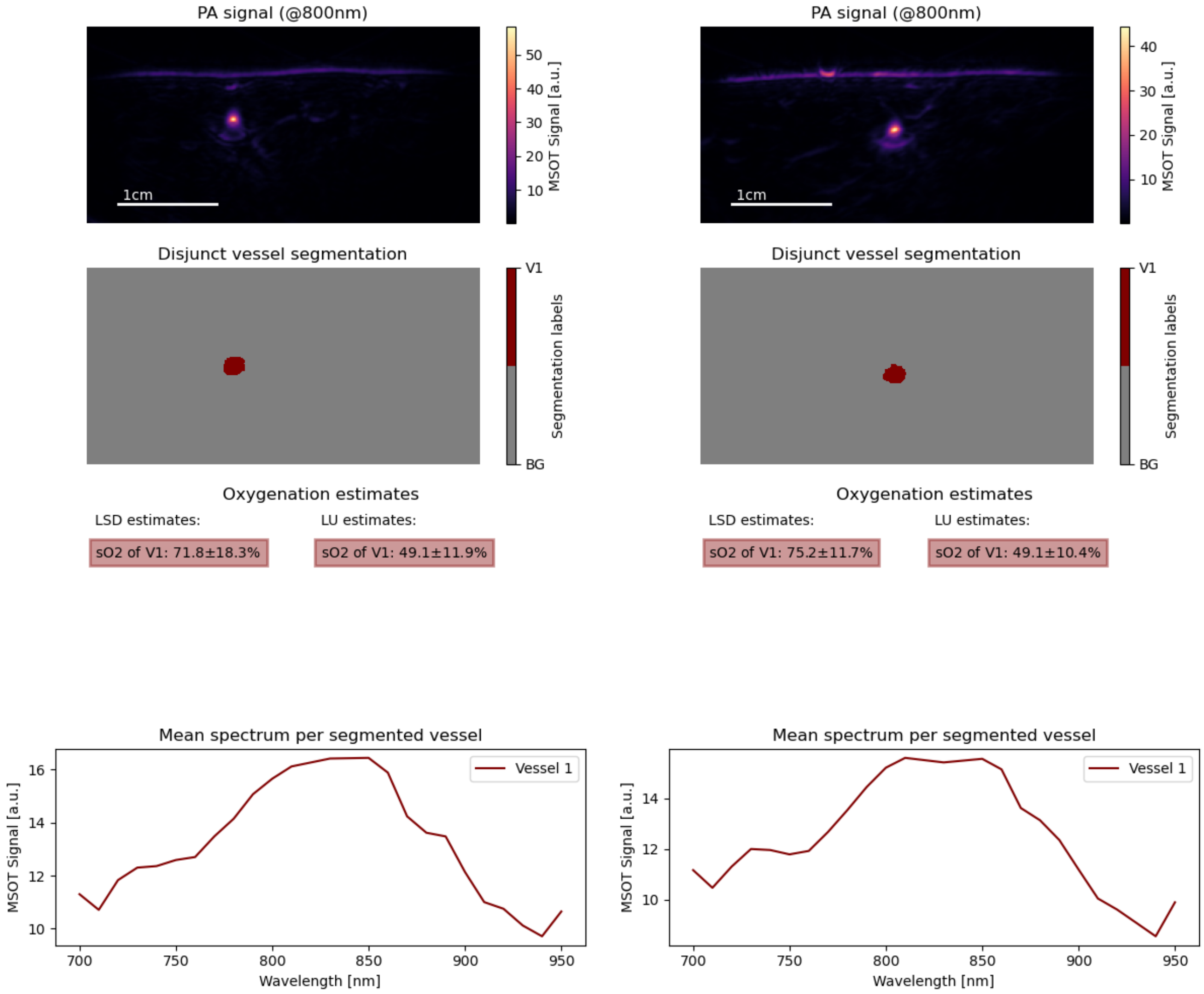

Figure 6. Result images from the human forearm data set.

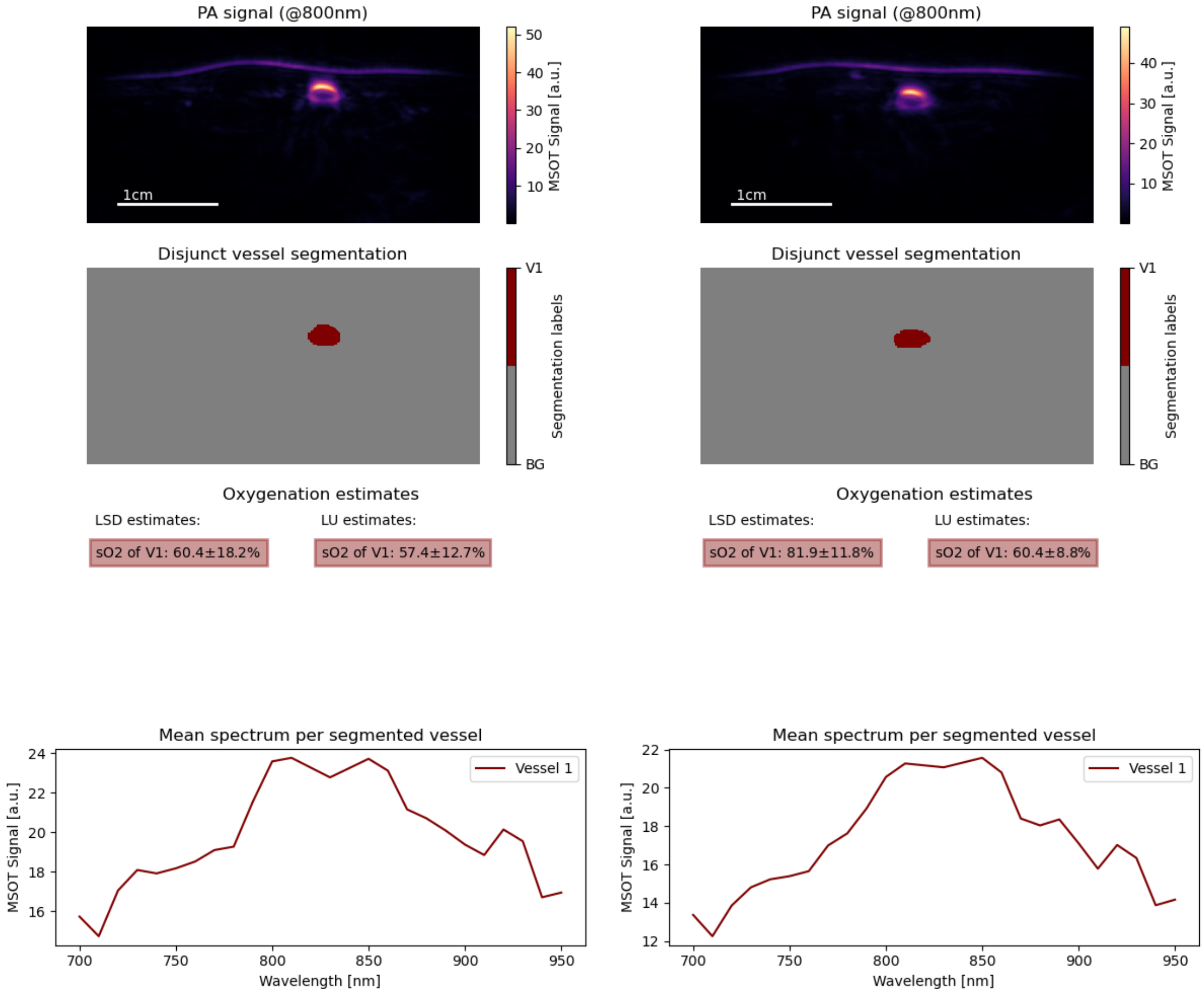

Figure 7. Result images from the human forearm data set.

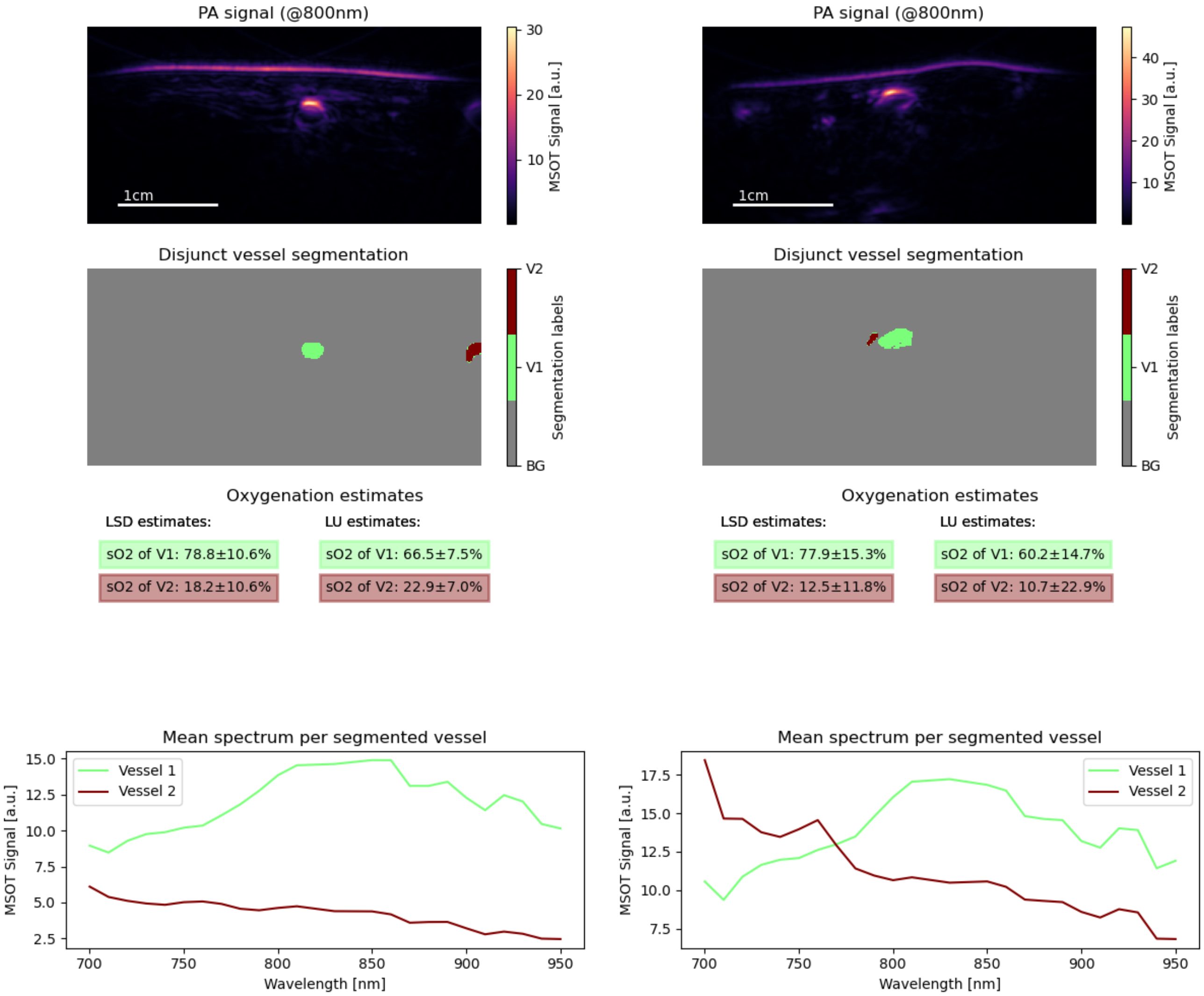

Figure 8. Result images from the human forearm data set.

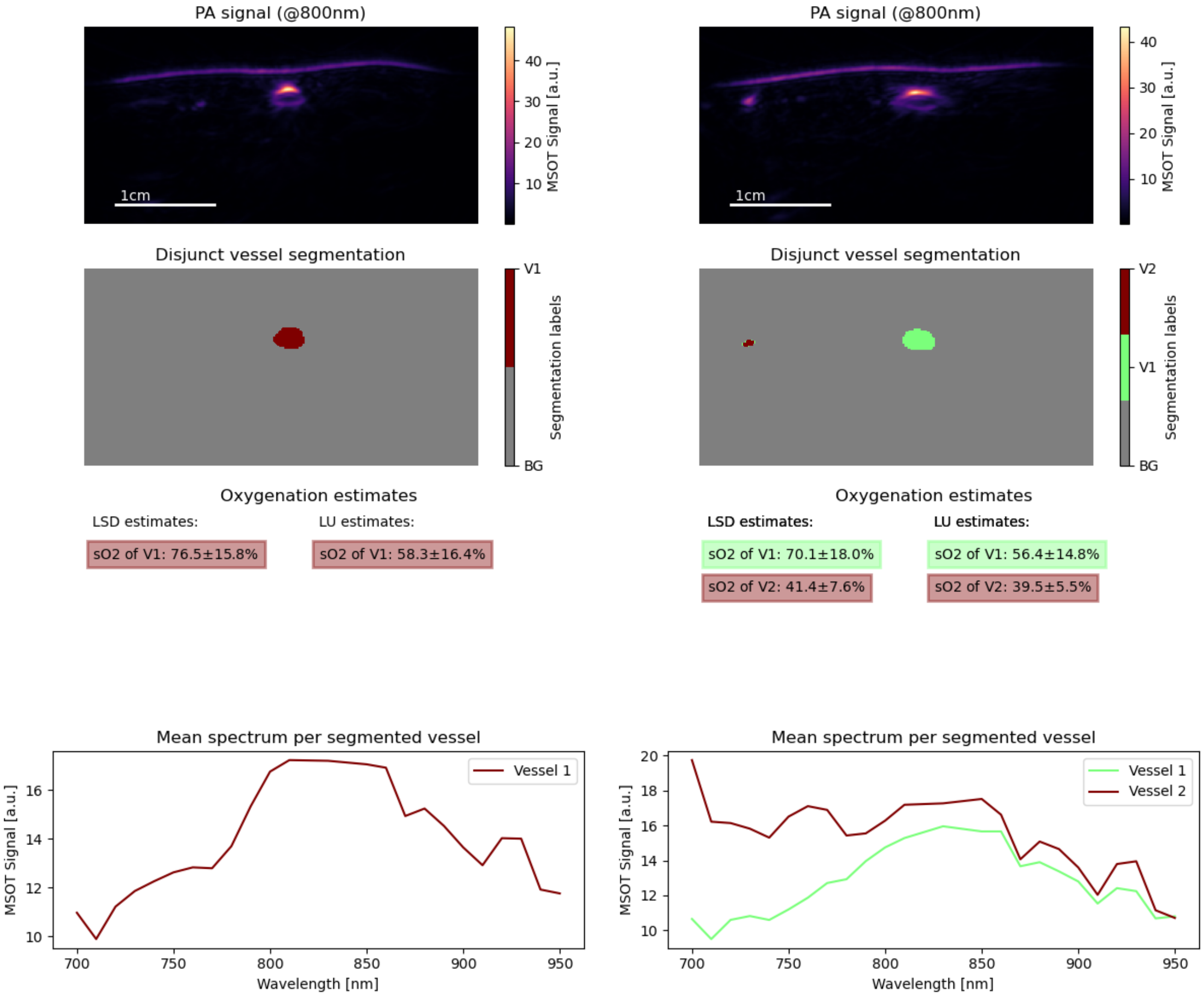

Figure 9. Result images from the human forearm data set.
